# Correlation between meniscal extrusion and symptom duration, alignment, and arthritic changes in medial meniscus posterior root tear: research article

**DOI:** 10.1186/s43019-019-0019-x

**Published:** 2020-01-01

**Authors:** Dong Hwi Kim, Gwang Chul Lee, Hyun Hak Kim, Dong Hyuk Cha

**Affiliations:** Department of Orthopaedic Surgery, College of Medicine, Chosun University Hospital, 365 Pilmundae-ro, Dong-gu, Gwangju, 61453 Republic of Korea

**Keywords:** Extrusion, Medial meniscus, Root tear, Chondral wear, Osteophyte, Alignment

## Abstract

**Background:**

Medial meniscus posterior root tear can result in medial meniscus extrusion. However, the severity of medial meniscus extrusion is different in each root tear patient. The purpose of this study was to identify the factors that contribute to the severity of medial meniscus extrusion with medial meniscus posterior root tear, such as duration of disease, the degree of arthritis—chondral wear, subchondral edema, osteophyte size, and Kellgren–Lawrence (K/L) grade—and mechanical alignment for appropriate treatment method.

**Methods:**

From January 2009 to August 2014, we retrospectively analyzed magnetic resonance imaging (MRI) and simple x-ray of 99 patients with medial meniscus posterior root tear. The duration of the disease was identified through retrospective chart review. The severity of medial meniscus extrusion, the presence of subchondral edema, the degree of chondral wear, and the size of the osteophyte were measured on MRI. K/L grade was confirmed on simple x-ray, and the mechanical axis was measured on whole extremity radiographs. Statistical analysis was performed by using bivariate correlation analysis and one-way analysis of variance.

**Results:**

The mean medial meniscus extrusion was 4.61 mm, and the mean duration of the disease was 15.52 months. The mean degree of chondral wear was 25.8%, and 63 out of 99 cases showed subchondral edema. The average alignment was 4.30 degrees, and the average size of the osteophyte was 1.48 mm. There were 40 cases (40.4%) with K/L grade I, 48 cases (48.5%) with grade II, 11 cases (11.1%) with grade III, and no cases with grade IV. In the group mean analysis between the K/L grade and the severity of medial meniscus extrusion, the average medial meniscus extrusions were 3.97 mm in grade I, 4.93 mm in grade II, and 5.59 mm in grade III. There was a statistical significance between the size of the osteophyte and the severity of medial meniscus extrusion (*P* = 0.000), K/L grade, and the severity of medial meniscus extrusion (*P* = 0.001).

**Conclusions:**

The severity of medial meniscus extrusion with medial meniscus posterior horn root tear is associated with the size of the osteophyte and K/L grade.

## Background

The meniscus has a function to protect the articular cartilage by reducing the distribution of load and stress on the joint surface by increasing the contact surface area between the femoral condyle and proximal tibial joint surfaces and plays an important role in lubrication [1–3]. The medial meniscus posterior horn is strongly attached to the tibial spine by the root and is the primary structure to maintain the hoop tension during loading [4]. The medial meniscus posterior root is rarely mobile and is particularly vulnerable to damage.

The medial meniscus posterior root tear leads to abnormal biomechanics of the tibiofemoral joint and the inability to convert axial loads into hoop stresses by inducing radial displacement of the medial meniscus, also called the medial meniscus extrusion [5]. Also, medial meniscus root tear causes increased contact pressure in the medial compartment, similar to the results of subtotal meniscectomy and root repaired knee is equal to the peak contact pressure on knee with intact medial meniscus [6]. Therefore, restoration of meniscal continuity is becoming the standard of care for posterior meniscal root pathology.

Medial meniscal extrusion is known to occur with degenerative meniscal tear or posterior root tear [7–9]. A medial meniscus extrusion of more than 3 mm has been linked to substantially increased articular cartilage loss and osteophyte formation [10]. The severity of the medial meniscus extrusion may also affect healing after repair, and several authors report that the medial meniscus extrusion is not completely reduced by medial meniscus posterior root tear repair [5]. However, in the case of the patient with medial meniscus posterior root tear, the severity of the medial meniscus extrusion was different, so we hypothesized that there could be factors affecting the severity of medial meniscus extrusion in addition to the medial meniscus posterior root tear. Factors such as duration of disease, the degree of arthritis (chondral wear, subchondral edema, osteophyte size, and Kellgren–Lawrence (K/L) grade), and mechanical alignment were assumed.

Lere et al. [11] and Puig et al. [12] studied the correlation between chondral damage and medical meniscus extrusion. Gale et al. [9] and Lee et al. [13] studied the correlation between osteophyte and medial meniscus extrusion. There is a great deal of research on the correlation between the medial meniscus extrusion and other factors, but there was no clarity. So we tried to investigate their relationship.

The purpose of this study is to investigate the factors that affect the severity of medial meniscus extrusion in medial meniscus posterior root tear on coronal plane magnetic resonance imaging (MRI), such as duration of disease, the degree of arthritis (chondral wear, subchondral edema, osteophyte size, and K/L grade), and mechanical alignment for appropriate treatment of the medial meniscus posterior root tear.

## Patients and Methods

One hundred thirty-seven patients who underwent arthroscopic treatment for the medial meniscus posterior root tear diagnosed by MRI at the authors’ institute between January 2009 and August 2014 initially enrolled in this study. Thirty-eight patients were excluded because of other associated injuries (lateral meniscus tear: 14, fracture: 3, other ligament injuries: 21). Consequently, 99 patients were finally included in this study. Of the 99 cases, 15 were males and 84 were females. The mean age was 58.57 (30~75) years. Sample size was calculated to be 84 patients using G power 3.0 program (expected correlation coefficient 0.30, threshold probability (type I error rate) 0.05, and power (1-type II error rate) 80%). So we selected 99 sample sizes that corresponded to the inclusion criteria. In the correlation analysis, when the absolute value of the Pearson correlation coefficient was 0.3 or more, the moderate correlation was shown. Therefore, the expected correlation coefficient was selected as 0.3.

Patients were assessed for the severity of medial meniscus extrusion, duration of the disease, presence of subchondral edema, degree of chondral wear, size of osteophyte, K/L grade, and mechanical alignment.

The duration of the disease was examined from the time of symptom onset to the time of MRI, and the mean duration was 15.52 (0~120) months.

**Fig. 1:**
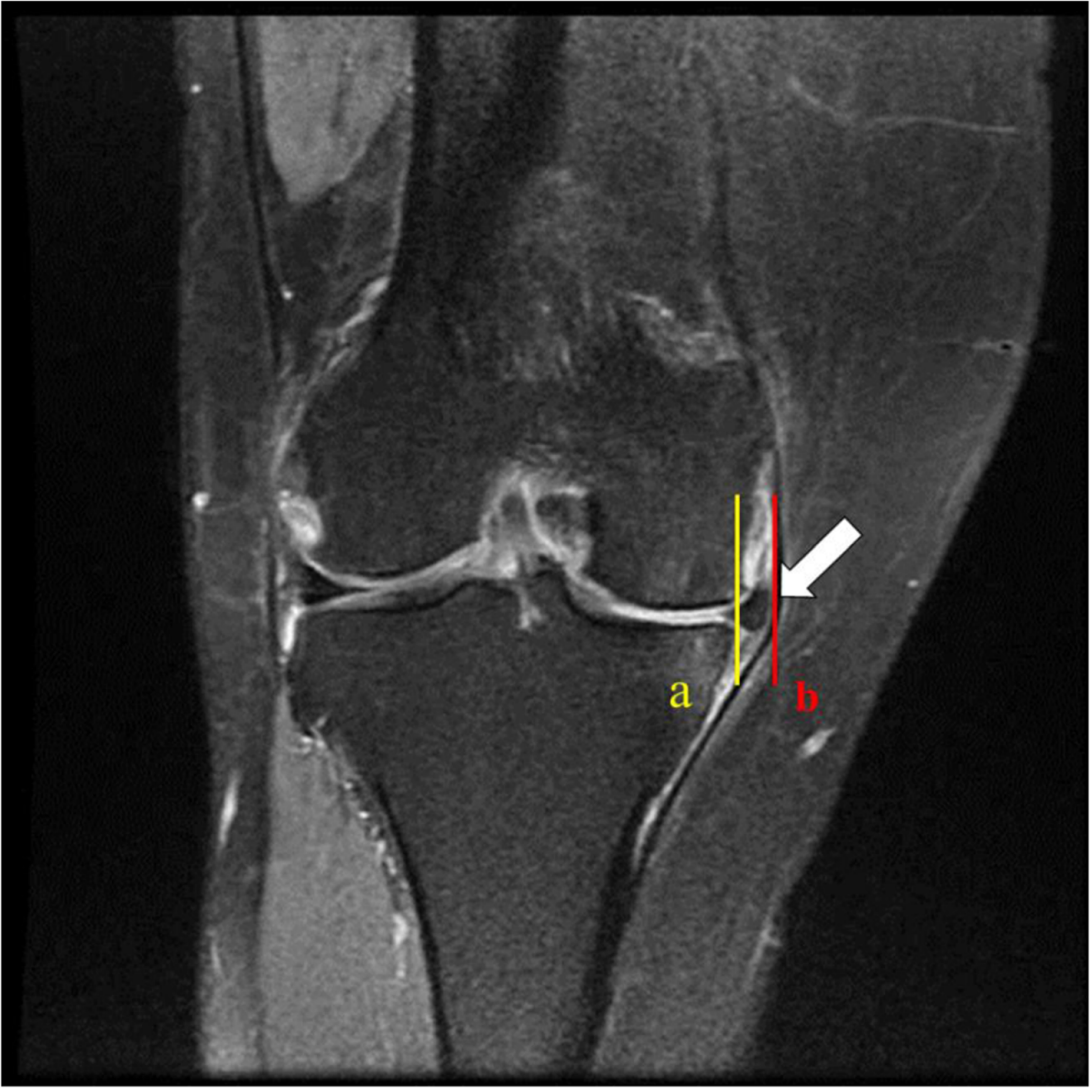
Measure the length between the vertical line (a) and the line (b), which represents the outer edge of the meniscus, in the medial side of the tibial proximal articular surface in the coronal plane magnetic resonance imaging, femoral medial condyle mid cut image

**Fig. 2:**
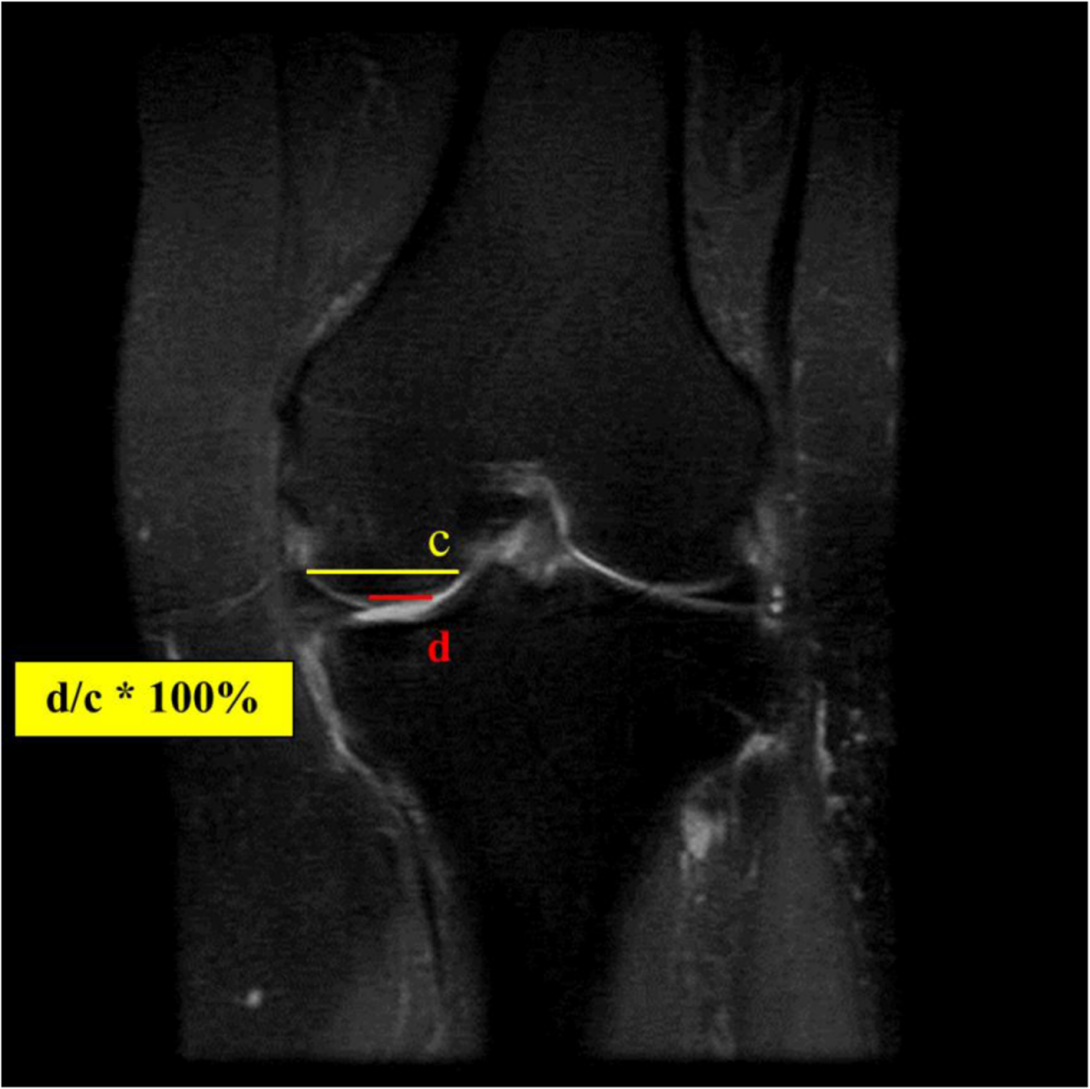
The size of the femur medial condyle (c) and the area with the greatest amount of chondral wear were measured as (d) and measured as d / c * 100%

**Fig. 3:**
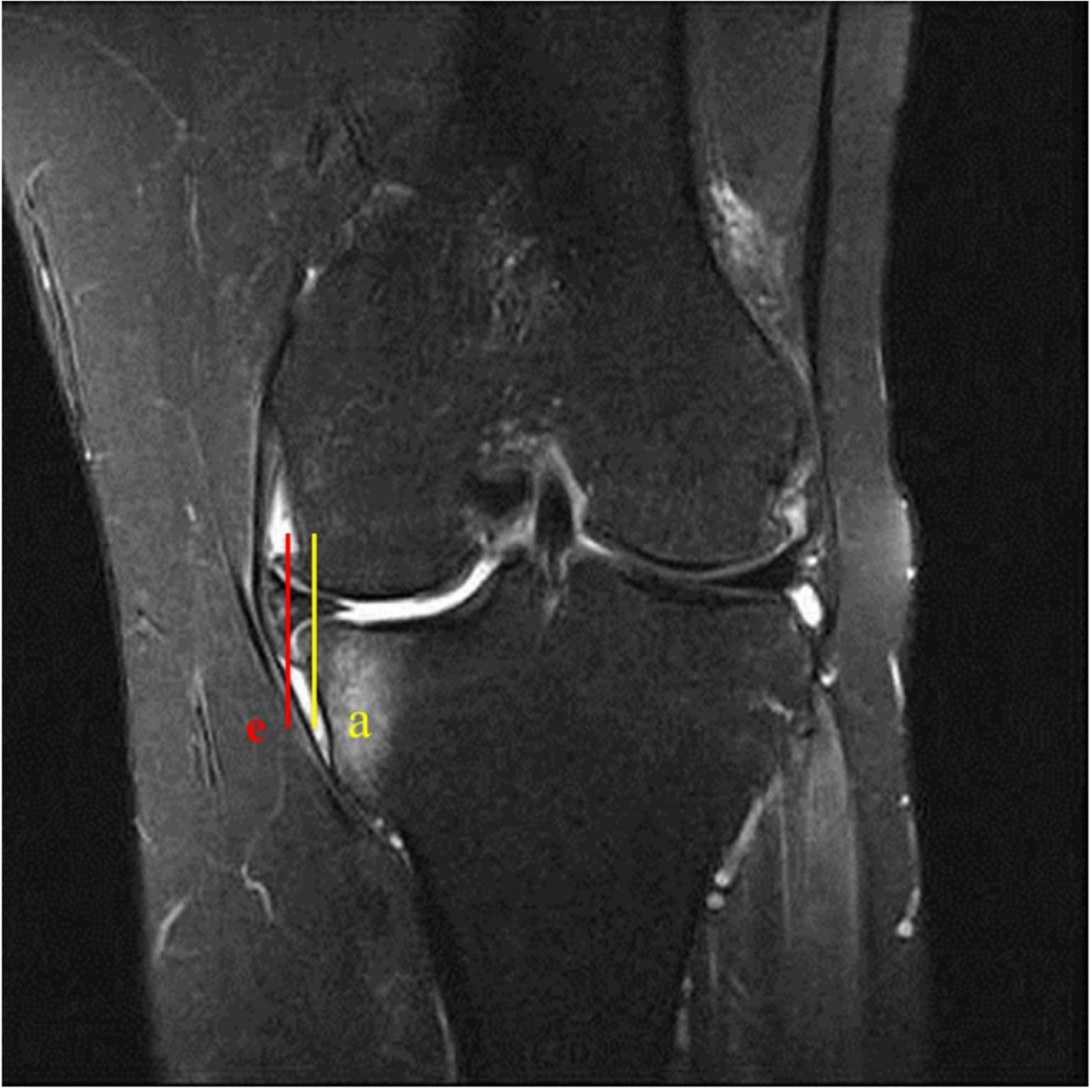
The distance between the line on the vertical line (a) and the line (e) representing the end of the osteophyte on the medial side of the tibial proximal articular surface

In the coronal plane MRI, the severity of the medial meniscus extrusion (Fig. 1), the presence of subchondral edema, the degree of chondral wear (Fig. 2) and the size of osteophyte (Fig. 3) were measured. The severity of the medial meniscus extrusion was measured by drawing a vertical line to the medial margin of the proximal tibia in the image of the middle part of medial femoral condyle of the coronal plane MRI and measuring the length from the vertical line to the outer edge of the medial meniscus. At this time, the osteophyte was excluded from the medial side of the proximal tibia.

The degree of chondral wear was measured as the percentage of chondral defects in the total length of the cartilage portion of the medial femoral condyle on the coronal MRI (Fig. 2). The size of the osteophyte was measured for the length grown from the medial end of the proximal tibia medial plateau on the same image measured the degree of extrusion of the medial meniscus (Fig. 3).

Radiographic evaluations were conducted by using five plain radiographs (weight-bearing whole leg anteroposterior (AP) view, weight-bearing AP view, 45° of flexion posteroanterior view, lateral view, and skyline view). All radiographic images were digitally acquired by using a picture archiving and communication system (PACS) (Impax; Agfa, Antwerp, Belgium), and assessments were subsequently carried out by using PACS software. Radiographs were evaluated by one of the authors blinded to the clinical information of the subjects at the time of reading and K/L grade were evaluated for the only medial compartment. K/L grades were defined as follows: grade 0, no features of osteoarthritis; grade 1, small osteophyte of doubtful importance; grade 2, definite osteophyte but an unimpaired joint space; grade 3, definite osteophyte with moderate diminution of joint space; and grade 4, definite osteophyte with substantial joint space reduction and sclerosis of subchondral bone. Lower limb alignment was assessed by measuring the mechanical tibiofemoral angle on whole leg standing AP radiographs. Mechanical tibiofemoral angle was defined as the angle formed by the intersection between the mechanical axes of the femur (the line from the femoral head center to femoral intercondylar notch center) and the tibia (the line from ankle talus center to the center of the tibial spine tips). A negative value was given to knees in valgus alignment.

All MRI was performed on an Avanto 1.5 Tesla MR (Siemens Medical Systems, Munich, Germany) using a phased array knee coil. A positioning device for the ankle and knee was used to ensure uniformity between patients. The MRI protocol for each subject included coronal, sagittal, and axial images. Coronal spin echo fat saturated proton density (repetition time (TR) 3990 echo time (TE) 36) and T2-weighed fat saturated images (TR 3180 TE 82) with a slice thickness of 3 mm, a 0.6-mm interslice gap, 1 AVERAGE, field of view 15 cm, and a matrix of 384 × 384 were used.

The collected data were analyzed by using the SPSS 18.0 statistical program and verified at a significance level of 5%. Statistical analysis was performed by using bivariate correlation analysis to investigate the correlation between severity of the medial meniscus extrusion, duration of disease, mechanical alignment, degree of chondral wear, and size of osteophyte. One-way analysis of variance (ANOVA) with post-hoc test (Scheffe) was performed to analyze the relationship between severity of the medial meniscus extrusion and K/L grade and the presence of the subchondral edema. Two orthopedic surgeons performed measurements twice on blinded radiographs with an interval of 2 weeks. Intraobserver and interobserver reliabilities for the assessment of radiographic measurements were tested by using single-measure intraclass correlation coefficients (ICCs) for the numerical values and kappa coefficients for the categorical values. An ICC of more than 0.8 was interpreted as reliable, and kappa of more than 0.6 was interpreted as a good strength of agreement.

3. ResultsThe ICCs for intra- and interobserver reliabilities ranged from 0.972 to 0.999, and kappa coefficients for intra- and interobserver reliabilities ranged from 0.848 to 0.978, which allowed us to have confidence in the reliability of the radiographic measurements produced by a single investigator.

On MRI examination, the mean medial meniscus extrusion was 4.61 (0~8.04) mm. The degree of the chondral wear was 25.8% (0%~70%) on the coronal MRI, and 63 out of 99 cases showed subchondral edema on MRI. The average size of the osteophyte was 1.48 (0~4.80) mm. On the standing whole leg AP view, the average mechanical alignment was 4.30 (−2.21 to 14.10) degrees. On plain radiographs, there were 40 cases (40.4%) of K/L grade I, 48 cases (48.5%) of grade II, 11 cases (11.1%) of grade III, and no grade IV cases.

**Fig. 4:**
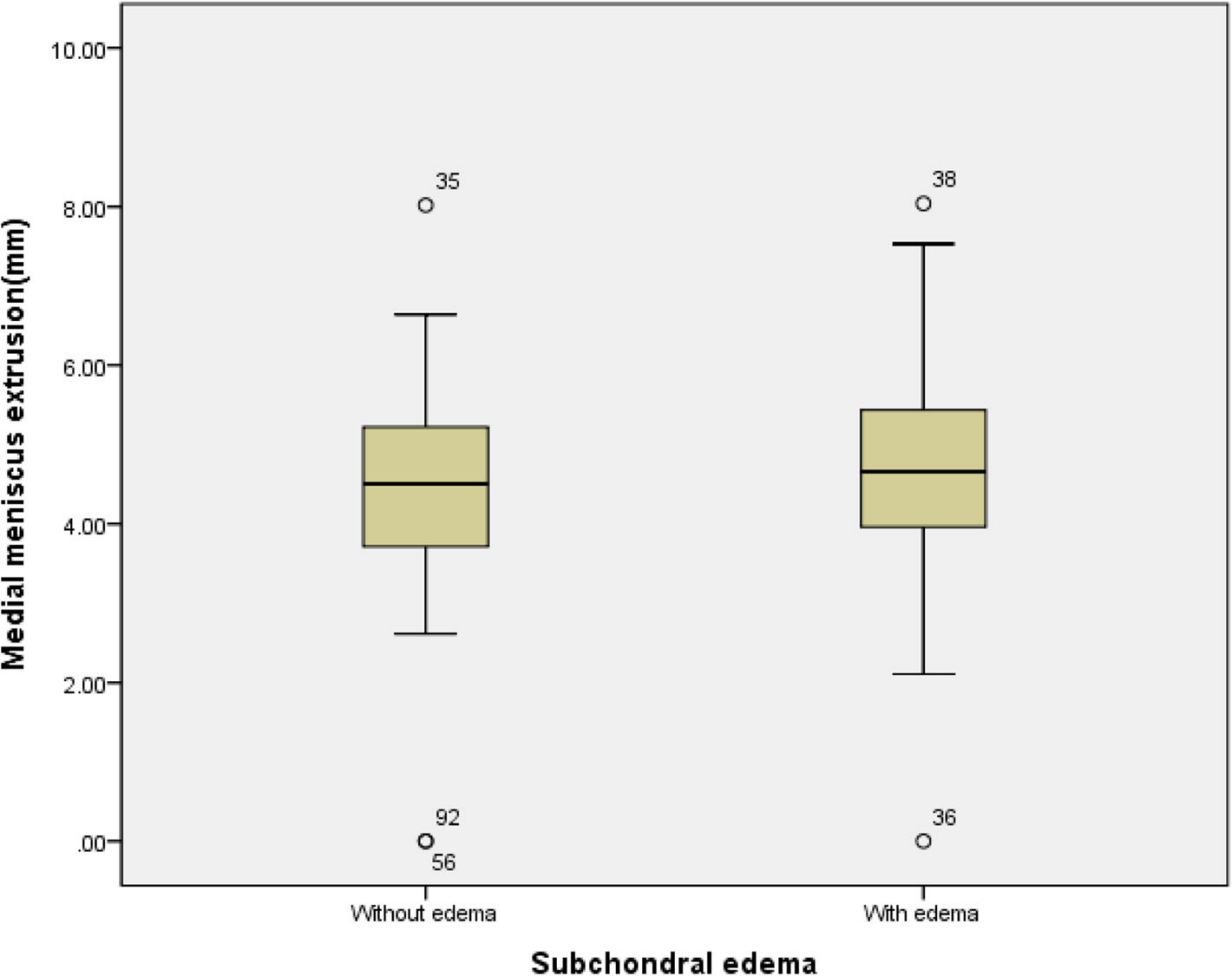
Group mean analysis between the presence of subchondral edema and the severity of medial meniscus extrusion

**Fig. 5:**
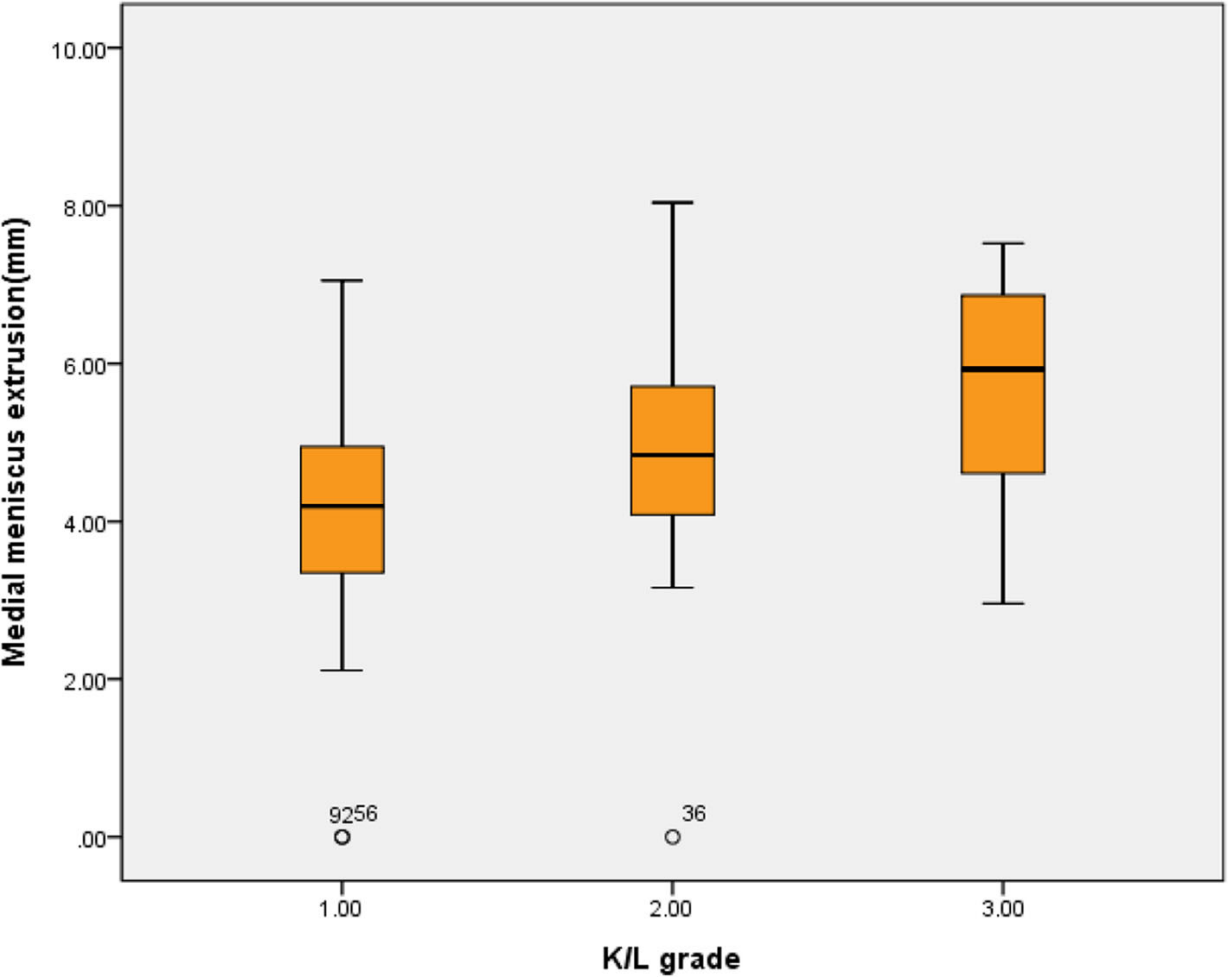
Group mean analysis between the Kellgren–Lawrence (K/L) grade and the severity of medial meniscus extrusion

There was no statistically significant relationship between the duration of the disease and the severity of the medial meniscus extrusion (*P* = 0.722) and also no statistically significant relationship between the mechanical alignment and the severity of the medial meniscus extrusion (*P* = 0.827). Chondral wear (*P* = 0.843) was also not statistically significant. There was statistical significance between the size of the osteophyte and the severity of the medial meniscus extrusion (*P* <0.001, Pearson correlation coefficient 0.530) (Table 1). According to the group mean analysis between the presence of subchondral edema and the severity of medial meniscus extrusion, the average medial meniscus extrusions were 4.35 mm (standard deviation (SD) 1.60) in the group without subchondral edema and 4.76 mm (SD 1.44) in the group with subchondral edema (Fig. 4). But there was no statistically significant relationship between the presence of subchondral edema and the severity of the medial meniscus extrusion (*P* = 0.195) (Table 2). In the group mean analysis between the K/L grade and the severity of medial meniscus extrusion, the average medial meniscus extrusions were 3.97 mm (SD 1.36) in grade I, 4.93 mm (SD 1.42) in grade II, and 5.59 mm (SD 1.51) in grade III (Fig. 5). There was statistical significance between the severity of the medial meniscus extrusion and K/L grade (*P* = 0.001) (Table 3).

**Table 1:** Correlation analysis between the severity of medial meniscus extrusion and other affecting factors

	Mean (range)	SD	CC^#^	*P* value^#^
Medial meniscus extrusion, mm	4.61 (0~8.04)	31.53		
Duration of disease, month	15.52 (0~120)	1.50	−0.036	0.722
Chondral wear, %	25.8 (0.0~70.0)	0.21	0.02	0.843
Mechanical alignment, °	4.3 (−2.21~14.1)	3.23	−0.022	0.827
Size of osteophyte, mm	1.48 (0~4.80)	1.24	0.513	0.000

^#^Statistical analysis was performed using the Pearson’s correlation analysis.

Abbreviations: *CC* correlation coefficient, *SD* standard deviation.

**Table 2:** Analysis of variance between the subchondral bone edema and medial meniscus extrusion

Subchondral bone edema	Mean, mm	SD	F value^#^	*P* value^#^
Without edema	4.35	1.60	1.706	0.195
With edema	4.76	1.44		

^#^Statistical analysis was performed using the one-way analysis of variance (ANOVA) analysis.

Abbreviation: *SD* Standard deviation.

**Table 3:** Analysis of variance between the K/L grade and medial meniscus extrusion

K/L grade	Mean, mm	SD	Scheffe	F value	*P* value^#^
I^a^	3.97	1.35	a > c	7.95	0.001
II^b^	4.92	1.42			
III^c^	5.58	1.51			

^#^Statistical analysis was performed using the one-way analysis-of-variance analysis.

Abbreviations: *K/L* Kellgren–Lawrence, *SD* standard deviation.

## Discussion

This study assessed the relationship between duration of disease, alignment, subchondral edema, degree of chondral wear, size of osteophyte, and K/L grade and the severity of the medial meniscus extrusion in posterior root tear. In our study, as the size of osteophyte and K/L grade increased, the severity of the medial meniscus extrusion increased.

Medial meniscus posterior root maintains the position of the normal meniscus [14] and plays an important role in preserving its function [15]. The meniscus without a strong attachment of the posterior horn root may tend to extrude to the medial side, which may affect the ability of the meniscus to absorb shock and load distribution [16]. The medial meniscus extrusion is highly correlated with the posterior horn root tear [15].

Femoro-tibial malalignment is a well-known risk factor for osteoarthritis of the knee [17]. In varus deformity, the load distribution may be further increased to the medial meniscus. Therefore, in this study, varus deformity was considered an aggravating factor affecting the medial meniscus extrusion in posterior root tear. Sugita et al. reported that the medial meniscus can be preserved well in a severe arthritic varus knee even in cases with already-diminishing medial compartment joint space [18]. In our study, there was no statistical significance between varus deformity and medial meniscus extrusion in posterior root tear. Malalignment can increase the load transmitted to the meniscus, which may lead to extrusion, but in posterior root tear, which has already lost hoop tension, malalignment did not affect extrusion more in our study.

Several studies have been performed to demonstrate the association between meniscus extrusion and chondral damage. An MRI study by Lerer et al. [11] showed a significant association between medial meniscus extrusion and chondral damage, and Puig et al. [12] found a significant correlation between arthroscopic findings and chondral damage when medial meniscus was extruded from MRI. But in this study, there was no statistically significant difference between medial meniscus extrusion. Lee et al. [13] assessed the association of arthroscopy-depicted chondral lesions and preoperative K/L grade with meniscal extrusion and found that only the K/L grade was significantly related to extrusion. Hellio et al. reported that meniscal damage may occur at the early stages of the osteoarthritis but that cartilage damage seems to appear later [19]. Based on this study, there may be a discrepancy between the timing of medial meniscus extrusion and chondral wear and thus no significant correlation. Posterior root tear of the medial meniscus can significantly increase the overall peak contact pressure in medial compartment and cause chondral wear, but the relationship between the medial meniscus extrusion and the chondral wear is not clear in our study.

The medial meniscus extrusion can cause premature osteoarthritis by reducing contact surface of femur and tibia, also increasing contact pressure [13]. In this study, we also investigated the relationship between subchondral edema, size of the osteophyte, and K/L grade in order to evaluate the association between medial meniscus extrusion and osteoarthritis. There was no statistical significance between the medial meniscus extrusion and the subchondral edema, but there was a statistically significant difference between the severity of medial meniscus extrusion and size of the osteophyte and the K/L grade. Lee et al. [13] reported that preoperative K/L grade had a greater effect on meniscal extrusion. Gale et al. [9] found that the amount of medial meniscus subluxation correlates with the degree of medial joint space narrowing. Although we identified statistical significance between the K/L grade and the severity of medial meniscus extrusion, we cannot affirm whether extrusion was a cause or consequence of osteoarthritis in this study.

Lee et al. [13] reported that the medial meniscus extrusion was associated with osteophyte of the joint and that the formation of osteophyte, which was a progressive change of osteoarthritis, can exacerbate the medial meniscus extrusion. In this study, the degree of meniscal extrusion was related to the increase in the size of the osteophyte, which can be understood as a natural result because the meniscus is connected to the tibial coronary ligament.

In this study, symptom duration was considered a factor affecting the medial meniscus extrusion degree, but there was no statistical significance between symptom duration and the medial meniscus extrusion (*P* = 0.722). Lim et al. reported that sudden onset of pain due to root tear resolved gradually within 3 months and clinical outcome was improved after non-operative treatment [20]. In addition, we thought that some patients may feel a pop sound in the meniscus root tear, but some patient may feel different, so it is difficult to determine the duration of the disease.

Limitations of this study are that it was retrospective, did not have a large sample. MRI scans should be performed to assess the presence and degree of meniscal extrusion in full weight-bearing. However, MRI is performed in a supine position with non-weight-bearing, so the degree of meniscus extrusion may be inaccurate. In addition, we may not have investigated other factors that may affect the medial meniscus extrusion. Also, it is unclear whether our results can reflect asymptomatic patients. Our study did not reveal any association between the severity of medial meniscus extrusion and the clinical outcomes of the patients.

## Conclusions

The severity of the medial meniscus extrusion with medial meniscus posterior root tear is associated with osteophytosis and K/L grade. Our results suggest that patients with osteophytosis, advanced K/L grade, and medial meniscus extrusion in posterior root tear may be carefully treated.

## Data Availability

Not applicable.

## Abbreviations

AP: Anteroposterior
ICC: Intraclass correlation coefficient
K/L grade: Kellgren–Lawrence grade
MRI: Magnetic resonance imaging
PACS: Picture archiving and communication system
SD: Standard deviation
TE: Echo time
TR: Repetition time
